# Histone H4 acetylation and the epigenetic reader Brd4 are critical regulators of pluripotency in embryonic stem cells

**DOI:** 10.1186/s12864-016-2414-y

**Published:** 2016-02-04

**Authors:** Michelle Gonzales-Cope, Simone Sidoli, Natarajan V. Bhanu, Kyoung-Jae Won, Benjamin A. Garcia

**Affiliations:** Epigenetics Program, Department of Biochemistry and Biophysics, Perelman School of Medicine, University of Pennsylvania, Philadelphia, PA 19104 USA; The Institute for Diabetes, Obesity, and Metabolism, Philadelphia, PA 19104 USA; Department of Genetics, Perelman School of Medicine, University of Pennsylvania, Philadelphia, PA 19104 USA

**Keywords:** Acetylation, Brd4, Embryonic stem cells, Epigenetics, Histone, Neuronal cells, Post-translational modifications, Mass spectrometry, ChIP-seq

## Abstract

**Background:**

Pluripotent cells can be differentiated into many different cell types in vitro. Successful differentiation is guided in large part by epigenetic reprogramming and regulation of critical gene expression patterns. Recent genome-wide studies have identified the distribution of different histone-post-translational modifications (PTMs) in various conditions and during cellular differentiation. However, our understanding of the abundance of histone PTMs and their regulatory mechanisms still remain unknown.

**Results:**

Here, we present a quantitative and comprehensive study of the abundance levels of histone PTMs during the differentiation of mouse embryonic stem cells (ESCs) using mass spectrometry (MS). We observed dynamic changes of histone PTMs including increased H3K9 methylation levels in agreement with previously reported results. More importantly, we found a global decrease of multiply acetylated histone H4 peptides. Brd4 targets acetylated H4 with a strong affinity to multiply modified H4 acetylation sites. We observed that the protein levels of Brd4 decreased upon differentiation together with global histone H4 acetylation. Inhibition of Brd4:histone H4 interaction by the BET domain inhibitor (+)-JQ1 in ESCs results in enhanced differentiation to the endodermal lineage, by disrupting the protein abundance dynamics. Genome-wide ChIP-seq mapping showed that Brd4 and H4 acetylation are co-occupied in the genome, upstream of core pluripotency genes such as Oct4 and Nanog in ESCs and lineage-specific genes in embryoid bodies (EBs).

**Conclusions:**

Together, our data demonstrate the fundamental role of Brd4 in monitoring cell differentiation through its interaction with acetylated histone marks and disruption of Brd4 may cause aberrant differentiation.

**Electronic supplementary material:**

The online version of this article (doi:10.1186/s12864-016-2414-y) contains supplementary material, which is available to authorized users.

## Background

Embryonic stem cells (ESCs) are self-renewing, pluripotent cells derived from the inner cell mass (ICM) of a blastocyst [1, 2]. Their ability to expand clonally or differentiate into multiple tissues makes them an ideal model to study cellular development. ESCs have been used for preclinical drug screening, toxicity testing, cell therapy in animal models and developing models for rare diseases, such as Fanconi anemia [3]. Epigenetic changes in histone and DNA methylation control cell commitment to a specific lineage [4, 5]. Therefore, understanding both the genetic and epigenetic mechanisms is important for successful regenerative therapy.

Histone post-translational modifications (PTMs) modulate the chromatin landscape by changing overall charge and by recruiting chromatin modifier enzymes, thereby facilitating gene expression/repression and DNA repair. Histone acetylation is associated with gene activation [6]. Loss of H4K16 acetylation at promoters results in decreased transcription [7]. While acetylated histones are found primarily at promoters of actively transcribed genes, they can also be found throughout the active gene [8]. Histone acetylation has been implicated to be important for the pluripotent cell state, as some HDAC inhibitors have been shown to improve reprogramming efficiency of induced pluripotent stem cells (iPSCs) or maintain a pluripotent stem cell state. For instance, when valproic acid, an HDAC inhibitor, is added only *Oct4* and *Sox2* are needed to reprogram MEFs [9]. Another HDAC inhibitor, butyrate, was also shown to improve reprogramming efficiency and can also reprogram myoblasts [10–12]. Since HDAC inhibition facilitates cellular iPSC reprogramming, histone acetylation may play an important role in pluripotency.

Histone acetylation is bound by bromodomain-containing proteins. Especially, acetylated histone H4 is bound by the double bromodomain proteins Brd2, Brd3, and Brd4, which possess histone chaperone activity, thus facilitating transcription through nucleosomes [13]. Brds have also been previously shown to play a role in development. Brd2 mutants are embryonic lethal, with Brd2 null mouse embryos showing deficient neural tube formation [14]. Similarly, Brd4 mutants are also embryonic lethal, and *in vitro* experiments show that they are incapable of maintaining the inner cell mass [15]. Recent study identified that deletion of *Brd4*, not *Brd2* and *Brd3*, disrupts the ESC colony formation in both human and mouse [16]. Brd4 also regulates ESC self-renewal and the expression of pluripotency genes [3, 16, 17]. Inhibition of Brd4 resulted in commitment to the neuroectodermal lineage [16]. In addition, Brd4 has been found to localize to and control the expression *HOX* gene clusters in HEK293 cells [18]. These double bromodomain containing proteins may have an important role both in ESC self-renewal and development.

Brd4 recruits the positive transcription elongation factor b (P-TEFb), which induces the release of the promoter-proximal paused RNA polymerase II [19]. Brd4 has also been shown to possess kinase activity and can bind the carboxy-terminal domain of RNA polymerase II to phosphorylate serine 2, which facilitates elongation by recruiting splicing factors [20]. Brd4 has been shown to facilitate transcription, after being docked by H3K9acS10ph/H4K16ac [21]. Recent crystal structures showed that while the second bromodomain of Brd4 binds most di-and tri-acetylated lysines, the first bromodomain of Brd4 (BD1) specifically binds di-, tri-, and tetra-acetylated histone H4 [22]. Another study using time-resolved fluorescence resonance energe transfer (TR-FRET) found that the strongest binding of BD1 was observed for the tetra-acetylated H4 peptide and the di-acetylated H4 peptide with K5 and K8, but weak binding was found for all mono-acetylated H4 peptide [23].

Even though histone PTMs and their readers are known to be important during cell differentiation, the changes in their abundance have not been previously well studied. In this work we investigated the changes in the quantity of histone PTMs during ESC differentiation into neuronal cells using quantitative mass spectrometry (MS) based proteomics. We found that the cell differentiation involves consistent rearrangement of global histone PTM abundance. In particular, we observed a reduction of doubly, triply and quadruply acetylated histone H4, suggesting an increase in chromatin compaction, and possible gene expression changes. Interestingly, the expression levels of Brd4, which recognizes multiply acetylated histone H4 N-terminal tails, were also reduced during differentiation. Inhibition of the binding of Brd4 to histone H4 using JQ1, a specific inhibitor for the Brd family in ESCs, promoted cell differentiation, while disrupting the coordinated changes in the quantity of histone PTMs. The genome-wide investigation using chromatin immunoprecipitation followed by sequencing (ChIP-Seq) against Brd4 and histone acetylation at H4 (H4ac) show that Brd4 binds to the pluripotent and lineage specific genes in embryoid body (EB) from ESCs, suggesting that inability of Brd4 to read H4 acetylation may disrupt genome-wide acetylation landscapes. Taken together, our results indicate that monitoring the epigenetic status through the interaction with acetylated histones is important for the maintenance of the pluripotency and the coordinated differentiation of ESCs.

## Methods

### Cell culture of mouse embryonic stem cells

CCE Nanog-GFP ESCs, a generous gift from Ihor Lemischka, were cultured in DMEM supplemented with 15 % FBS, Penicillin-Streptomycin, non-essential amino acids, 2-mercaptoethanol, sodium pyruvate, and ESGRO/2i (Life Technologies, Carlsbad, CA, USA), as adapted from Gaspard et al. [24]. Cells were seeded on gelatin-coated tissue culture dishes at 2x10^6^ cells per 10 cm dish and passaged every other day. Cells were grown at 37°C in a humidified 5 % CO_2_/95 % air incubator. *Nanog*-GFP was measured by FACS (Wistar Institute Flow Cytometry Facility, Philadelphia, PA, USA) prior to experiments to verify that >90 % of cells were pluripotent. No ethics approval was required for this study.

### Retinoic acid differentiation

To differentiate ESCs, we applied the protocol described earlier [25]. To form embryoid bodies, ESCs were plated on petri dishes in ESC media containing 10 % FBS and lacking ESGRO/2i. Cells were incubated on a shaker at 30 rpm for 4 days, changing media on alternate days. Embryoid bodies were grown for four more days in EB media containing 1 μM all-*trans* retinoic acid (Sigma-Aldrich, St. Louis, MO, USA), changing media also on alternate days. RA-treated EBs were plated on gelatin-coated tissue culture dishes and incubated for a week, changing media every other day.

### Nuclei isolation and histone extraction

Nuclei were isolated and histone proteins were extracted as described before [26]. Briefly, histones were acid-extracted from nuclei with 0.2 M H_2_SO_4_ for 2 hours and precipitated with 33 % trichloroacetic acid (TCA) overnight. Samples were resuspended in 20-30 μl of ddH_2_O and protein concentration was calculated using the Bradford assay. Histone samples were derivatized with propionic anhydride, digested with trypsin overnight at 37C and re-propionylated as previously described [26]. Afterwards, histone peptides were desalted using C_18_ material.

### Reversed phase nano chromatography coupled to mass spectrometry

Samples were analyzed by using a nanoLC-MS/MS setup. nanoLC was configured with a 75 μm ID × 17 cm Reprosil-Pur C18-AQ (3 μm; Dr. Maisch GmbH, Germany) nano-column using an EASY-nLC nanoHPLC (Thermo Scientific, Odense, Denmark). The HPLC gradient was 2-35 % solvent B (A = 0.1 % formic acid; B = 95 % MeCN, 0.1 % formic acid) over 30 min and from 34 % to 100 % solvent B in 30 minutes at a flow-rate of 300 nL/min. LC was coupled with an LTQ-Orbitrap Velos mass spectrometer (Thermo Fisher Scientific, San Jose, CA). Full scan MS spectrum (m/z 290 − 1400) was performed in the Orbitrap with a resolution of 60,000 (at 400 m/z) with an AGC target of 1x10e6. The acquisition method contained both data-dependent and targeted scans. The targeted signals were the histone H3 and H4 peptides in isobaric forms, if any. MS/MS was performed with collision induced dissociation (CID) with normalized collision energy of 35, an AGC target of 10e4 and a maximum injection time of 100 ms. MS/MS data were collected in centroid mode. Precursor ion charge state screening was enabled and all unassigned charge states as well as singly charged species were rejected. All proteomics data has been deposited in the Chorus database (https://chorusproject.org/pages/dashboard.html#/projects/all/1013/experiments).

### Histone PTM data analysis

The selected modified peptides were quantified using label-free based extracted ion chromatography. EpiProfile was used for the purpose, with a peak extraction mass tolerance set to 10 ppm [26]. The peptide relative ratio was calculated by considering the peak area of all peptides that share the same amino acid sequence as total peptide abundance, and estimating the percentage of each individual species by dividing the peak area by the total peptide abundance.

### Western blot analysis

Cells were lysed using TNE buffer (50 mM Tris-HCl, 100mM NaCl, 0.1 mM EDTA). Approximately 10 μg of cell lysate per sample were run on a 10 % polyacrylamide gel. The membrane was incubated with the primary antibodies (anti-Brd4: Abcam 128874, anti-Lamin-B1: Abcam 133741, anti-βIII-tubulin: Abcam 18207, anti-Oct4: Abcam 19857, anti-acetyl H4: Millipore 06-866) according to the manufacturers’ instructions overnight at 4°C. To determine the sensitivity of detection of the anti-acetyl H4 antibody (Millipore 06-866), a dot blot was performed. 0.2 μg, 0.4 μg, 0.8 μg, and 1.2 μg of H4 peptides from the Acetyl-Histone Peptide Pack (Upstate) were spotted onto a nitrocellulose membrane. 1.5 μg, 3.0 μg, 6.0 μg, and 12.0 μg of HPLC purified histone H2B from HeLa and ESCs were also spotted onto the membrane. The membrane was incubated with the primary antibody at a 1:2,000 dilution for 30 minutes at room temperature. The membrane was incubated with secondary antibody at a 1:10,000 dilution for 30 minutes at room temperature (Pierce 31463) then probed with ECL Western Blot Substrate (Pierce).

### Gene expression analysis

Cells were homogenized using QIAshredder (Qiagen), and RNA was isolated using an RNeasy Kit (Qiagen). cDNA was synthesized using a Superscript III First-Strand Synthesis System (Invitrogen). qPCR was performed using *Power* SYBR® Green PCR Master Mix (Applied Biosystems) and the StepOne Real-Time PCR System (Applied Biosystems) according to manufacturer’s instructions. The qPCR primers used are listed in Additional file 1: Table S1. Primers for GAPDH, Oct4, Nanog, Gata4, COL2A1 and Brd4 were designed using Universal Probe Library Assay Design Center (Roche), while the rest were published earlier [19, 27, 28]. Relative mRNA abundance to GAPDH was calculated by DDCt method.

### Chromatin Immunoprecipitation-Sequencing (ChIP-Seq)

Approximately 4x10^7^ CCE ESCs and embryoid bodies were hypotonically lysed in TMSD buffer (40mM Tris pH7.5, 5mM MgCl_2_, 250mM sucrose, 1 mM DTT) containing inhibitors (500 μM AEBSF, 5 μM microcystin, 10 mM sodium butyrate). Nuclear pellets were resuspended in 1 mL micrococcal nuclease digestion buffer (10mM HEPES pH7.5, 50mM NaCl, 5mM CaCl_2_, 5mM MgCl_2_) containing inhibitors. Micrococcal nuclease (Sigma) was added to each sample (3 units/1 mL sample) and incubated at 37°C for 30 minutes. The digestion was quenched by the addition of 20 mM EGTA. Samples were pelleted, and the resulting supernatant was collected. The pellet was washed twice with BC50 (40mM Tris pH7.5, 5mM MgCl_2_, 50mM NaCl, 5 % glycerol) containing inhibitors, and the resulting supernatants were pooled with the first supernatant. Mononucleosomes were immunoprecipitated from the supernatant overnight at 4°C using Protein AG magnetic beads (Pierce) bound to either anti-Brd4 antibody (Abcam 128874) or anti-acetyl H4 antibody (Millipore 06-866). The magnetic beads were washed three times with BC50, then resuspended in ~50uL BC50. The magnetic beads were treated with RNase A (Roche) followed by Proteinase K (Denville) according to the manufacturers’ instructions. The samples were then cleaned up with DNA Clean & Concentrator-5 (Zymogen). The ChIP library was prepared using the multiplexed ChIP-Seq sample preparation protocol available on the University of Pennsylvania Functional Genomics Core website (http://fgc.genomics.upenn.edu/). ChIP libraries were run on a 2 % agarose gel to verify amplification and size. ChIP libraries were submitted to SeqMatic (Fremont, CA) for Illumina HiSeq sequencing. Sequencing data were sent to BioInfoRX (Madison, WI) for analysis using BxChIPSeq 2.0. Briefly, bowtie [29] was used to align raw sequence reads to the mouse genome (mm9) with options ‘-v 2-m 1 --best --strata,’ and all of the redundant tags were removed before downstream analysis. Peak calling was carried out using HOMER [30] with a default option (FDR = 0.001 and Poisson *p*-value cutoff = 0.0001) on ChIPed samples against the matching input samples. Since the two replicates for each ChIP had similar profiles, the data were combined to make a single peak call. All ChIP-Seq data were normalized to 10 reads per million mapped reads (RPM). A specific peak was defined as having an enrichment within a 200 bp region of more than 4-fold between two cell populations. The remaining peaks were defined as constitutive. Gene ontology (GO) analysis was performed by using GREAT [31]. All genomics data have been deposited in the GEO repository (GSE76760).

### BET domain inhibition

ESCs were grown in differentiation conditions in ESC media, and cells were treated with 100nM or 200nM (+)-JQ1 (Abcam) for 4 days, with media changes on alternate days. DMSO was used as a control.

## Results

### Global changes of the abundance of histone methylation and acetylation during ESC differentiation

**Fig. 1:**
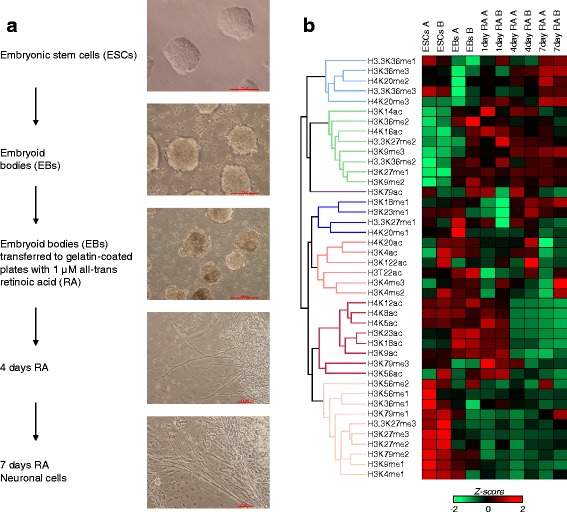
Cell morphology and dynamic changes in abundance of histone PTMs. **a** Retinoic acid differentiation of ESCs results in neuronal cells. From top to bottom, CCE ESCs were grown in suspension for 4 days in the absence of LIF/2i to form embryoid bodies. The embryoid bodies were treated with 1 μM all-trans retinoic acid for 4 days. Cells were plated on gelatin-coated plates and grown for 4 days and 7 days. Red scale bar represents 100 μm. **b** Relative abundance of histone PTMs during cell differentiation. Heatmap of all single histone modifications from histone H3 and H4, calculated by summing the relative abundance of all peptides carrying each given PTM. A and B represent the two biological replicates for each analyzed time point. The represented value was obtained by averaging the three technical replicates and performing z-score normalization for the rows

ESCs were differentiated to embryoid bodies (EBs) by culturing them in suspension. EBs were further differentiated using all-*trans* retinoic acid (RA). Microscopy images were taken at each time point that cells were collected: ESCs, EBs, RA-treated EBs, and RA-treated cells plated for 4 and 7 days (Fig. 1). Cells showed evident morphological changes at each stage, with the appearance of filamentous projections in the plated RA-treated cells, consistent with neuronal differentiation using RA. To identify the changes of histone PTM abundance during ESC differentiation, histones were isolated from cells collected at each time point and analyzed by mass spectrometry (MS) using our Bottom Up MS platform consisting of chemical derivatization and high-resolution MS [32].

The MS analysis provided us with the changes in global abundance of histone methylation and acetylation (Fig. 1b and Additional file 2: Figure S1). The analysis using principle component analysis (PCA) confirmed that the histone PTM abundance profiles at ESCs are distinguishable to those at EBs and RA-treated cells (Additional file 2: Figure S1A). Statistical analysis using one-way ANOVA identified the histone PTMs whose quantities are significantly affected during differentiation. For instance, H3K9me2 increased (*p*-value: 5.6e-10) soon after differentiation into EB, consistent with the increase of the genome-wide distribution of H3K9me2 upon cell differentiation [33], while H3K9me1 abundance become reduced upon differentiation (*p*-value: 8.8e-13) (Additional file 2: Figure S1B). Also, H3K4me1 levels were significantly reduced upon differentiation among other K4 methylation (17.1 % of the total histone H3 in ESCs became 13.0 % in day 7 differentiated neuronal cells; *p*-value: 1.6e-5) (Additional file 2: Figure S1), which also well resonant to the recent genome-wide survey of H3K4me1 in various cell types [34]. H3K27me3 drastically reduced from ESCs to day 7 (from 25.9 % to 7.1 %; *p*-value: 7.8e-26), which does not match with the genome-wide survey where H3K27me3 levels were increased in the normalized intergenic H3K27me3 signals [34]. The discrepancy with this study may due to the focal distribution of H3K27me3 in ESCs, which expands upon differentiation [34, 35]. Abundance of H3K36 methylation was also affected especially in EBs, with 44.4 % H3K36me2 compared to 30.8 % in ESCs, which may indicate the emergence of mixed cell populations and non-neuronal lineages consistent with spontaneous differentiation (Additional file 2: Figure S1B).

The majority of histone acetylation marks become significantly reduced in their quantity upon RA treatment including H3K23ac, H4K8ac, H4K12ac and H4K5ac. On the other hand, H3K14ac and H4K16ac were increased after differentiation (Additional file 2: Figure S1C), showing dynamic changes in the abundance in histone acetylation as well as methylation.

### Decrease of multiply acetylated lysine on H4 during differentiation

**Fig. 2:**
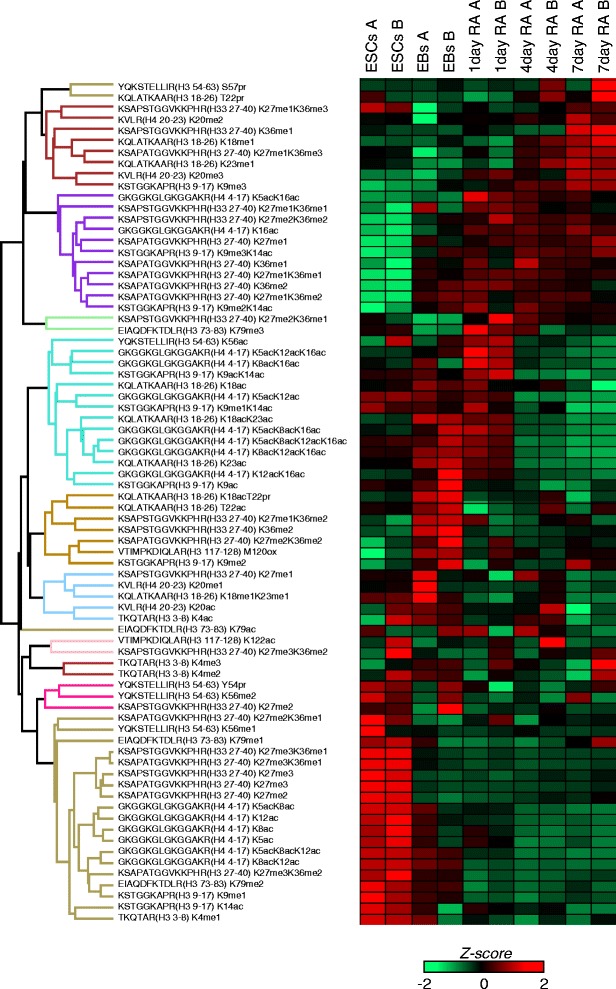
Histone peptide regulation during differentiation. Heatmap of all quantified peptides using nLC-MS from histone H3 and H4. A and B represent the two biological replicates for each analyzed time point. The represented value was obtained by averaging the three technical replicates and performing z-score normalization for the rows.

Unique to our approach is the ability to quantify multiple histone PTMs. For instance, we observed that H3K27me3K36me2 was reduced (Fig. 2) while the relative quantity of H3K36me2 was increasing during the differentiation (Fig. 1b). Especially, using the intact peptide GKGGKGLGKGGAKR (histone H4, aa 4-17), which contains all the histone H4 acetylation sites, we could also investigate the co-existence of up to four histone acetylation marks on lysine residues. We observed unique changes of the combinatorial histone PTM abundance. Interestingly, multiply acetylated peptides including tetra-acetylated peptide (K5, K8, K12, K16) were decreased during differentiation. Indeed, majority of the peptides with multiply-acetylated lysine were decreased in the differentiated cells compared with ESCs except for H4K5acK16ac (Fig. 2). This may in part be due to H4K16ac increasing upon differentiation (Fig. 1b). In this context, is interesting to note that Brd4 binds to all multiply acetylated H4 sites except H4K16ac, coupling histone PTMs to transcriptional elongation [36, 37].

### Brd4 is a marker for self-renewal that binds to multiply acetylated histone proteins

**Fig. 3:**
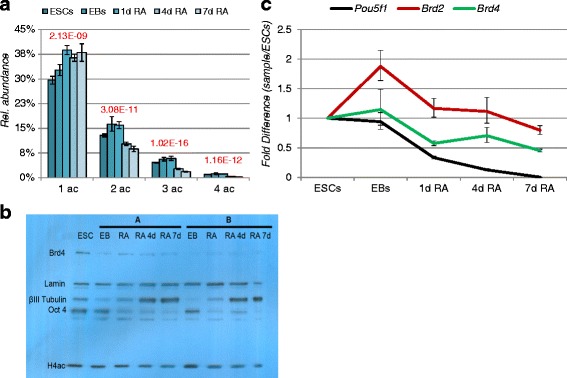
Regulation of histone acetylation and Brd4. **a** Analysis of the acetylated state of the peptide of histone H4 aa 4-17. The singly acetylated form increases during differentiation, while the hyperacetylated states decrease. Above the bars in red the ANOVA *p*-value (one tail test) among the different conditions is displayed. **b** Western blotting of Brd4, Oct4 βIII-tubulin and Lamin, this last one used as control. Brd4 decreases during differentiation, and so Oct4. On the bottom, antibody recognizing acetylated histone H4. **c** qRT-PCR of *Oct4* (*Pou5f1*, in black), *Brd2* (in red) and *Brd4* (in green) across the five time points of the differentiation process. *Actin* was used as the reference gene

The decreased abundance in multiply acetylated lysine prompted us to further investigate the abundance of mono-and multiply acetylated peptides using histone H4 aa 4-17 peptide. We observed that this peptide containing two, three and four acetylations decreased significantly during differentiation, while monoacetylated H4 peptides increased (Fig. 3a). As multiply acetylated histone PTMs are the potential targets for Brd4, we investigated the changes of the Brd4 abundance during differentiation. Western blot analysis of the ESCs, EBs and RA-treated cells revealed that the pluripotency master regulator Oct4 decreased as RA differentiation progressed, while neuronal marker βIII tubulin increased. In line with down-regulation of Oct4, the protein levels of Brd4 also decreased upon EB formation and further in differentiated cells in the two replicates we investigated (Fig. 3b). The decreasing levels of histone H4 acetylation observed in mass spectrometry was not very obviously confirmed in Western analysis as the antibody was raised to tetra-acetylated H4. However, a slightly decreasing trend of acetylated H4 can be seen as ESCs differentiate.

Cells collected at each time-point were also analyzed by qRT-PCR for expression of *Oct4 (Pou5f1), Brd2 and Brd4* (Fig. 3c). EBs were grown for 4 days in suspension; RA after 4 days grown in suspension with RA. RA 4d and RA 7d were grown on gelatin coated plates (Fig. 3c). *Oct4* levels were maintained in EB, indicating lingering presence of *Oct4* transcripts. On the other hand, Oct4 decreased rapidly upon differentiation in the presence of RA which is a strong differentiation agent. Importantly, *Brd4* levels decreased upon RA differentiation, synchronous with *Oct4* expression, further indicating that their expressions are linked each other. We also investigated the expression of another related BET domain protein, *Brd2*. We found that in general, the expression of *Brd2* also somewhat mirrored the overall *Brd4* and *Oct4* expression trend, to the extent of showing a spike in the EB stage, similar to Oct4. This may explain increase of some acetylation marks at EB stage. However, the changes of its levels in the differentiated cells compared with ESCs are not significant. Besides, previous study found that Brd2 does not disrupt the self-renewing characteristics of ESCs [16]. Taken together, all this data suggest that histone acetylation and Brd4 are most likely the major epigenetic determinants of ESC pluripotency.

### Effect of BET domain inhibition on the epigenome and cellular pluripotency

**Fig. 4:**
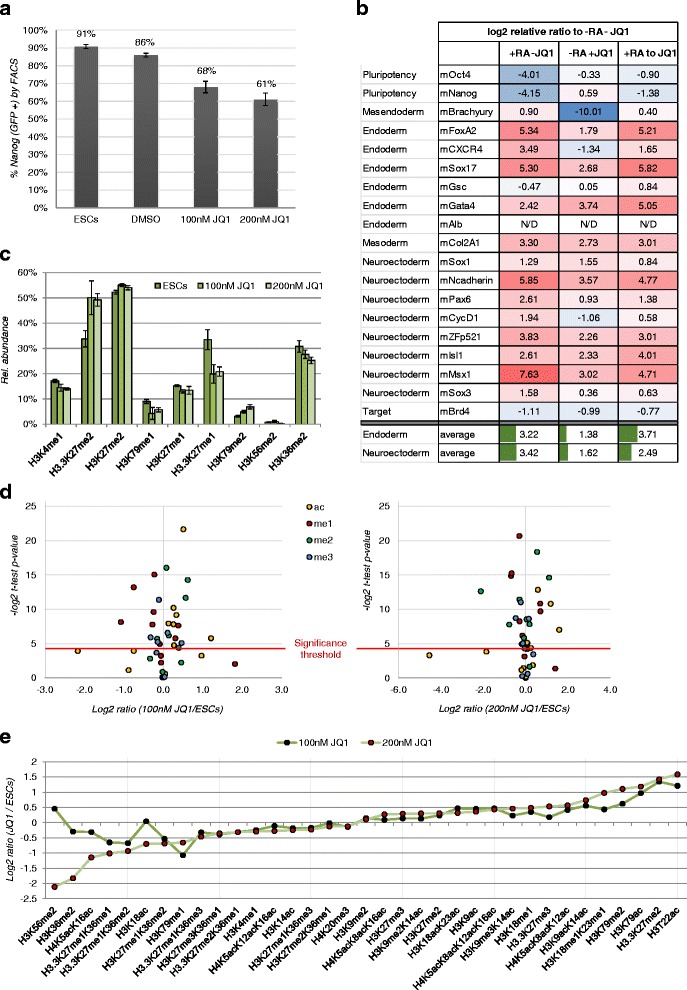
Analysis of key regulatory proteins and histone PTMs during JQ1 cell treatment. **a** (+)-JQ1 treatment of ESCs results in a decrease in Nanog-GFP as measured by FACS. **b** In triplicate experiments, ESCs were initiated into differentiation in medium lacking LIF. Matched cultures comprising DMSO control (-RA-JQ1), RA treatment for 6 days (+RA-JQ1), JQ1 treatment for 6 days (-RA + JQ1) and RA for 3 days followed by JQ1 for 3 days were maintained and for 6 days with media changes on alternate days. On harvest, mRNA abundance was analyzed by qPCR using a panel of primers (Additional file 1: Table S1). The DDCt data was represented as relative fold over DMSO control (-RA-JQ1). Expression of pluripotency markers, *Oct4* and *Nanog* decreases upon RA and (+)-JQ1 treatment as compared to DMSO control without drug, while expression of germ layer markers increased. The trends of differentiation by inhibition of BET domain by these two treatments and a combination thereof are shown as averages (bottom cells) of multiple markers, respectively for endoderm and neurectoderm. **c** Most significant histone PTM changes during inhibitor treatment. **d** Representation of all histone PTMs by plotting their log2 fold change between untreated cells and treated with 100 nM (left) or 200 nM (right) of JQ1 inhibitor. Significant changes were considered when the–test *p*-value (homoscedastic, two tails) was <0.05, equivalent to >4.32 when transformed into–log2. Color code represents different histone modification types. **e** Representation of all histone peptide regulations in cells untreated vs treated with JQ1. The two different doses were marked with black and red dots. The figure displays a tight correlation of histone peptide regulation between the two treatments

In order to confirm the role of Brd4 during differentiation, ESCs were treated with either 100 nM or 200 nM of the BET domain inhibitor (+)-JQ1 for 4 days (Fig. 4a). Since the ESCs were engineered to express *Nanog*-GFP, we assessed pluripotency by FACS as Nanog is only expressed in pluripotent cells. About 91 % of the ESCs were *Nanog*-GFP positive, while it was about the same in DMSO (86 %) As expected, (+)-JQ1-treated ESCs showed a decrease in *Nanog*-GFP: about 68 % expressing *Nanog*-GFP following 100 mM (+)-JQ1 treatment and 61 % following with 200 mM (+)-JQ1 treatment (Fig. 4a).

For further analysis of gene expression subsequent to BET inhibition, the expression level of markers for endoderm, mesoderm and neuroectoderm in the following conditions were analyzed: ESC (Day 0), DMSO control (-RA-JQ1), 1 μM RA treatment for 6 days (+RA-JQ1), 2 μM JQ1 treatment for 6 days (-RA + JQ1) and RA for 3 days followed by JQ1 for 3 days (+RA to JQ1). All differentiation conditions were devoid of LIF. In order to compare the relative mRNA expressions ranging from 0.00003946 to 1, the data was normalized using z-score and was represented as relative fold over DMSO control (-RA-JQ1) (Fig. 4b). As expected, all the three treatment conditions downregulated expression of pluripotency genes, *Oct4* and *Nanog. Brachyury*, a mesodermal marker seems to be either unchanged or very low compared to DMSO control. Surprisingly, there was induction of both endoderm and neurectoderm markers with all treatment regimens. However late endoderm marker specific for hepatocytes, *Alb* (albumin) was not detected. Average expression of all endodermal markers was similar to that of neurectodermal markers (3.22 vs 3.42) with RA treatment, while with JQ1 alone they were confirmed that JQ1 promoted differentiation comparable to RA. The potency of RA and JQ1 is validated by inhibition of Brd4 expression in all treatment conditions. However, unlike earlier reported, we did not find JQ1 augmenting the neurectodermal induction by RA when treated with in succession (+RA to JQ1) [28].

In addition, histones were analyzed and compared to DMSO-treated ESCs. Histone modifications also were found to change in their relative abundance upon (+)-JQ1 treatment (Fig. 4c, d, e and Additional file 3: Figure S2). As we predict JQ1 to promote differentiation, we noted that both concentrations of (+)-JQ1 provided very comparable effects on the fold changes of histone PTMs as compared to ESCs control (Fig. 4d, e). BRD4 was reported to bind to enhancers with H3K4me1 [38] and in this study, JQ1 decreased H3K4me1 (Fig. 4c). The vast majority of acetylated marks were found to be regulated (Fig. 4c and Additional file 3: Figure S2). Acetylation such as H4K16ac that are decreased in differentiation, were also decreased upon JQ1 treatment, indicating that JQ1 treatment had no effect on this mark. This is in agreement with H4K16ac not being the binding partner of BRD4 [39]. Similarly, tetra-acetylated peptide (K5, K8, K12 and K16) increased upon the JQ1 treatment, suggesting that decrease of the tetra-acetylation is not a requirement for cell differentiation. On the other hand, H3K79ac and K9ac were increased on BRD4 inhibition. Moreover, di-acetylated peptide (K5 and K16) and tri-acetylated peptide (K5, K12 and K16) were significantly reduced. These show that JQ1 facilitates cell differentiation, but the epigenetic landscapes it changes are rather broad and should only be interpreted in a locus-specific manner. Collectively, our data suggest that differentiation of ESCs is tightly linked to failure of Brd4 binding to chromatin, which alters the global epigenome.

### Re-distribution of Brd4 biding site during differentiation

To understand the role of Brd4 in pluripotency, we performed ChIP-Seq against Brd4 and the highly acetylated state of histone H4. We first confirmed the specificity of both the Brd4 (Abcam 128874) and acetyl-H4 antibodies (Millipore 06-866). The Brd4 antibody normally detected a 250 kDa protein (Additional file 4: Figure S3). For detecting acetyl-H4, we dot blotted 0.2 μg-1.6 μg of H4 peptides (individual monoacetylated peptides and a tetraacetylated peptide), as well as nuclear extract from HeLa and ESC cells. Hybridization of anti-acetyl H4 to H4 (residues 1-20) peptides (K5ac, K8ac, K12ac and K16ac) revealed that among the singly acetylated H4 peptides, the antibody slightly recognizes H4 acetylated at K5, strongly recognizes H4 acetylated at K8 and at K12, but does not recognize H4 acetylated at K16 (Additional file 4: Figure S3). More importantly, the antibody also strongly recognizes the tetra-acetylated H4 peptide, acetylated at all four lysine residues (K5, K8, K12 and K16). It does not recognize the unmodified H4 peptide, which was the biggest concern for non-specific binding. Due to the similar nature of histone H2B tails, HPLC-purified H2B from HeLa and ESCs were tested as well. The anti-acetyl H4 antibody has some slight cross-reactivity with H2B, an unfortunate, but unavoidable small issue.

**Fig. 5:**
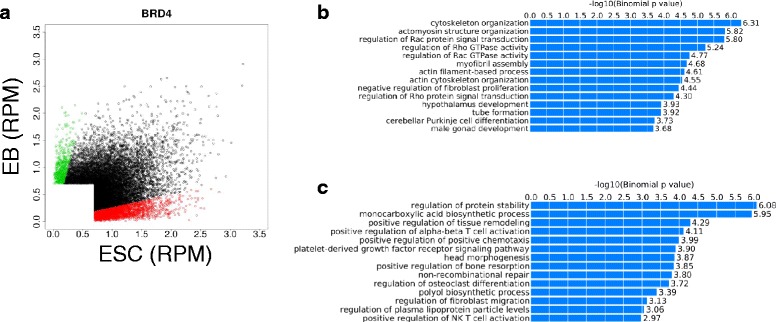
Redistribution of Brd4 binding sites during differentiation. **a** ChIP occupancy for ESC (red) and EB (green) specific Brd4 peaks. Read per million (RPM) was calculated for the Brd4 occupancy in ESC and EBs (**b**) GO for EB specific peaks. Terms related with neuronal differentiation are enriched (**c**) GO analysis for the ESC specific peaks

To understand the role of Brd4, we further investigated genomic distribution of Brd4 in ESCs and EBs. Applying the Homer peak calling algorithm, we identified 2,754 ESC specific, 567 EB specific and 11,128 peaks constitutive to both cell types (Fig 5a). Gene ontology (GO) analysis using GREAT found that the enriched terms for the EB specific peaks include “hypothalamus development” and “cerebellar Purkinje cell differentiation”, suggesting that Brd4 gained binding close to the genes related with neuronal development in EBs (Fig 5b). On the other hand, the enriched terms for the ESC specific peaks include “head morphogenesis” and “regulation of osteoclast differentiation”, suggesting that loss of Brd4 for the developmental genes other than neuronal development in EBs (Fig. 5c). The constitutive peaks are enriched for the terms such as “regulation of intrinsic apoptotic signaling pathway”, “blastocyst formation”, and “histone H4-K5 acetylation” (Additional file 5: Figure S4).

**Fig. 6:**
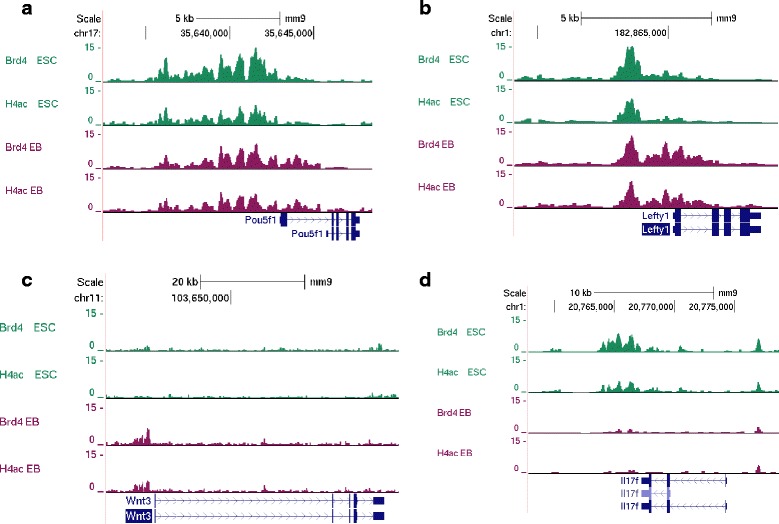
Genes associated with H4ac and Brd4 binding. Brd4 and H4ac occupancies in ESCs and EBs are shown. **a**) Constitutive Brd4 peaks near *Pou5f1* (**b**) EB specific increase in Brd4 occupancy at *Leafy1* promoter (**c**) EB-specific Brd4 peaks at *Wnt3* promoter (**d**) ESC specific Brd4 peaks at *Il17f* promoter

*Oct4* (*Pou5f1*), a marker for pluripotency showed constitutive Brd4 peaks between ESCs and EBs (Fig 6a). The promoter region of left-right determination factor 1 (*Lefty1)*, which is important for axis development [40], also have the constitutive peaks, but its Brd4 levels are stronger in EBs (Fig 6b). Wnt3, important factor for neuronal development [41] also gained a *Brd4* peak at its promoter region (Fig 6c). IL-17F, an important factor for T cells, lost the *Brd4* peak in ESCs, These well represent that EBs have mixed population of stem cells and already committed cells and *Brd4* reflects the characteristics of cells. Cell-type specific *Brd4* peaks match well with the enrichment of H4ac (Fig, 6 and Additional file 6: Figure S5), showing the high affinity of Brd4 with H4ac. Brd4 overlapped with more than 20 % of pluripotent factors (*Oct4*, *Sox2*, and *Nanog*) and more than 30 % of *c-Myc* and *n-Myc* binding sites within 1Kbps [42]. However, the overlap with an insulator *CTCF* is only 6 percent. These show that Brd4 is involved in transcriptional mechanism of diverse TFs, but not with insulators (Additional file 7: Table S2).

## Discussion

Brd4 among other BET domain family members uniquely characterizes ESC maintenance [3, 16, 17]. We investigated the role of Brd4 using proteomic as well as genomic approaches during differentiation and sought functional validation by treating with the Brd4 inhibitor, JQ1. Our proteomic approaches revealed the changes in the abundance of histone PTMs, which were dynamically regulated during the ESC differentiation. For instance H3K4me1, a mark associated with enhancers for active transcription [43] decreased during differentiation in this study and could well be used to localize genes that were turned on in the RA-directed ESC differentiation model by ChIP-Seq. On the other hand, this study confirms earlier reports of depletion of H3K27me3 during differentiation [44]. Besides these and other significant changes in many acetylated sites, we quantified histone PTMs such as H3K18me1 and H3K23me1 that have not been well studied previously. The current data embodies evidence for unknown roles for these PTM marks during differentiation.

Changes in global acetylation levels were marked during differentiation with RA and JQ1. One example is global reduction of H3K9acK14ac. This agrees with the previous work indicating that H3K9ac and H3K14ac co-localize with bivalent domains (H3K4me3 and H3K27me3) in ESCs [45]. Therefore, the decrease of H3K9acK14ac may be linked to the loss of bivalent domains upon differentiation. Moreover, H3K9me2K14ac and H3K9me3K14ac are significantly increased in differentiated cells compared to ESCs. This is not surprising since H3K9me2 and H3K9me3 have been shown to be a barrier to pluripotency of reprogrammed induced pluripotent stem cells [46, 47].

Majority, except for H3K14ac and H4K16ac, of the acetylated peptides were decreased in differentiation, indicating that acetylation marks may be differentially regulated during differentiation. Interestingly, tetra-acetylated peptides in H4 (K5, K8, K12 and K16) were reduced in their abundance during differentiation. These multiply acetylated H4 peptides are the major target for Brd4. As noted earlier, H4K16ac, which increased in its abundance during differentiation is not the binding partner of Brd4 although Brd4 binds to many other kinds of histone acetylation [39].

Brd4 is the double bromodomain protein that binds hyperacetylated histone H4 and promotes passage of elongating RNA polymerase II through activating transcribed genes. There was a correlation between decrease in Brd4 abundance and histone H4 hypoacetylation (Fig. 3a and c) in differentiating ESCs. This was further mirrored in Brd4 inhibition studies where the BET domain inhibitor (+)-JQ1 counteracts and inhibits Brd4 binding to target chromatin sites in ESCs and caused the ESCs to differentiate as determined by decreased *Nanog*-GFP expression, and by qRT-PCR for pluripotency and differentiation*.* It is conceivable that Brd4 activates these genes by binding to acetylated histone H4.

Western blotting confirmed that the pluripotency factor Oct4 decreased upon differentiation, while the neuron-specific βIII tubulin protein increased. Concomitantly, Brd4 levels decreased immediately upon EB formation and further with RA induced differentiation. Moreover, qRT-PCR analysis performed on *Oct4*, *Brd2*, and *Brd4* showed that while both *Oct4* and *Brd4* expression decreased upon differentiation, while *Brd2* expression did not show the same immediate trend. This suggests that *Brd2* and *Brd4* have potential different functions in pluripotency and differentiation. In agreement with these results, a study recently showed that Brd4 depletion impairs ESC colony formation while Brd2 depletion has no such effect [3]. The decrease of Brd4 with differentiation led to the next question of whether inhibition of Brd4 could also induce cellular differentiation.

Inhibition of Brd4 by (+)-JQ1 treatment of ESCs was associated with corresponding down-regulation of the pluripotency genes, *Nanog* and *Oct4*, presumably through inhibited Brd4. These observations are consistent with previous work demonstrating Brd4 regulation of *Nanog* and *Oct4* expression [3, 16]. The induction of neurectodermal lineage was evidenced by the concomitant increase in the expression of panel of markers (*Sox1, NCadherin, Pax6, CycD1, ZFp521, Isl1, Msx1 and Sox3*). Besides, RA also induced endoderm and this effect is well-documented. RA is a morphogen that has been used in in vitro pancreatic differentiation protocols for inducing early pancreatic markers [27, 48]. Elsewhere, studies have reported the induction of neurectoderm over endoderm [3, 19]. Similarly, RA has been shown to potentiate mesoderm in ES cells in the presence of other morphogens such as BMP [49]. However, induction of these germ layers by JQ1-effected Brd4 inhibition resulted in an increase in H3K9 methylation, which is known to be associated with differentiation [50]. Further, Brd4 peaks overlapped with H4ac enrichment in our ChIP-Seq analysis. These experiments indicate that Brd4, histone H4 acetylation and active transcription presumably of master developmental genes (i.e. *Oct4*) are linked, and the genome-wide localization of Brd4 confirms its involvement in pluripotency.

Contrary to the global decrease of H4 tetra-acetylation upon the JQ1 treatment, we identified global increase of acetylation histone forms. This indicates that the epigenetic landscape becomes disrupted after JQ1 treatment. The genome-wide re-distribution of Brd4 strongly indicates that Brd4 maintains self-renewal as well as play a role during differentiation in concert with acetylated histone PTMs. The loss of the Brd4 function by JQ1 may prevent cells from monitoring the levels of acetylated histones. The loss of monitoring function may responsible for the increase of tetra-acetylation upon JQ1 treatment.

## Conclusions

Collectively, our data demonstrate that Brd4 plays a fundamental role in regulating cell differentiation with its binding to acetylated histones. Specifically our inhibitor treatment study proves that disruption of Brd4 interaction to acetylated histone marks may cause aberrant differentiation.

### Availability of supporting data

All proteomics raw mass spectrometry data files has been deposited in the Chorus database (https://chorusproject.org/) under the identifier 1013. All genomics data may be accessed from this web link portal: http://projects.bxgenomics.com/Results/Seqmatic_ChIP_wR5Ybh7icZ/Report/ChIP_Seq_Results2014_Oct_20.html.

## Additional files

Additional file 1: Table S1. Primer sequences for qPCR analysis w/o RA and JQ1 treatment (from Fig. 4B) (XLSX 9 kb) Additional file 2: Figure S1. (A) Principal component analysis (PCA) of the ten samples analyzed. The yellow oval marks the initial time point, i.e. ESCs, while the blue oval represents the final stage of cell differentiation. (B) Statistical changes for the major methylated sites of histone H3 across all stages of differentiation. The *p*-value, represented in parenthesis for each PTM, was calculated by performing ANOVA (one tail). (C) Most significant changes of histone H3 and H4 acetylations. The–Log10 of the ANOVA *p*-value was represented in boxes above the bar plot (significant if >1.30, equivalent to the–Log10 of <0.05). (PPTX 96 kb) Additional file 3: Figure S2. Histone peptide regulation during JQ1 treatment. Heatmap of all quantified peptides using nLC-MS from histone H3 and H4 in ESCs and ESCs treated with JQ1 inhibitor. (PPTX 461 kb) Additional file 4: Figure S3. Validation of antibody against histone H4 acetylation and ChIP-Seq of key chromatin binding proteins. Dot blot testing the specificity of anti-H4 acetyl antibody. Specific amounts of various H4 peptides and histone H2B were dotted onto the membrane. Antibody strongly recognizes H4 peptide singly acetylated at K8 or K12, and tetra-acetylated H4 (K5, K8, K12, K16). (PPTX 183 kb) Additional file 5: Figure S4. GO analysis for the peak constitutive for both ESCs and EBs. (PPTX 711 kb) Additional file 6: Figure S5. Heatmap for ESC and EB specific Brd4 binding sites. Brd4 binding well matches with the enrichment for H4ac. (PPTX 526 kb) Additional file 7: Table S2. The overlapping peaks of Brd4 with TFs in ESCs within 1Kbps. (XLSX 10 kb)

## Abbreviations

ChIP-Seq: chromatin immunoprecipitation followed by next generation sequencing
DMSO: dimethyl sulfoxide
EBs: embryoid bodies
ESCs: embryonic stem cells
HDAC: histone deacetylase
iPSCs: induced pluripotent stem cells
MS: mass spectrometry
MS/MS: tandem mass spectrometry
PTMs: post-translational modifications
qPCR: quantitative PCR
qRT-PCR: quantitative reverse transcription PCR
RA: retinoic acid
