# Supplementary material for: Histone H4 acetylation and the epigenetic reader Brd4 are critical regulators of pluripotency in embryonic stem cells
Source: BMC Genomics. 2016 Feb 4;17:95. doi: 10.1186/s12864-016-2414-y (PMC4740988; doi:10.1186/s12864-016-2414-y)
Supplement: Additional file 2: Figure S1. — (A) Principal component analysis (PCA) of the ten samples analyzed. The yellow oval marks the initial time point, i.e. ESCs, while the blue oval represents the final stage of cell differentiation. (B) Statistical changes for the major methylated sites of histone H3 across all stages of differentiation. The p-value, represented in parenthesis for each PTM, was calculated by performing ANOVA (one tail). (C) Most significant changes of histone H3 and H4 acetylations. The–Log10 of the ANOVA p-value was represented in boxes above the bar plot (significant if >1.30, equivalent to the–Log10 of <0.05). (PPTX 96 kb) [file 12864_2016_2414_MOESM2_ESM.pptx]

## Slide 1
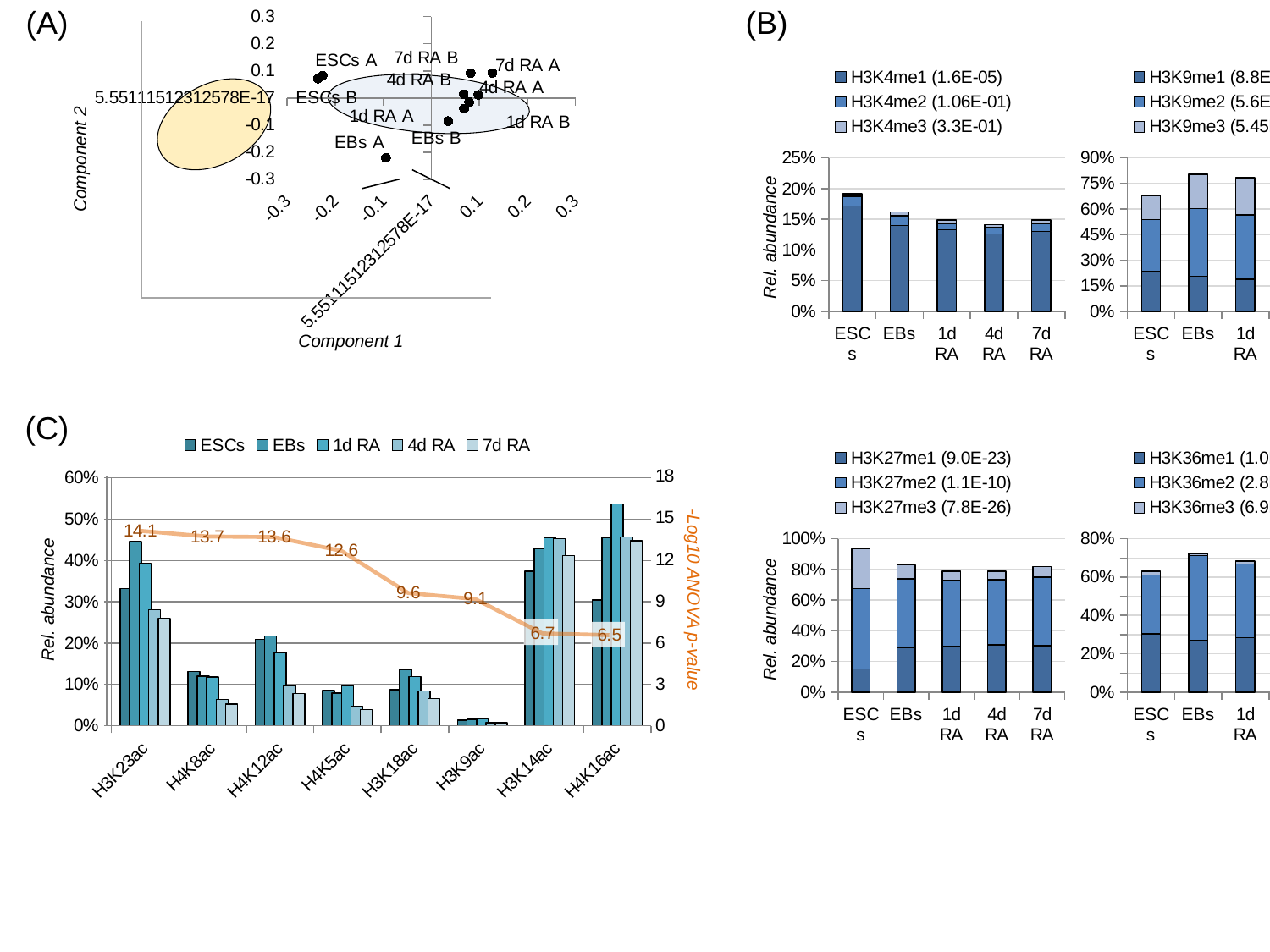

### Chart
| Category | ESCs A | ESCs B | EBs A | EBs B | 1d RA A | 1d RA B | 4d RA A | 4d RA B | 7d RA A | 7d RA B |
|---|---|---|---|---|---|---|---|---|---|---|(A)
(B)
### Chart
| Category | H3K4me1 (1.6E-05) | H3K4me2 (1.06E-01) | H3K4me3 (3.3E-01) |
|---|---|---|---|
| ESCs | 0.171530833333333 | 0.0162061666666667 | 0.00371433333333333 |
| EBs | 0.139724 | 0.016195 | 0.00612016666666667 |
| 1d RA | 0.133034666666667 | 0.0105958333333333 | 0.00511916666666667 |
| 4d RA | 0.125835833333333 | 0.0108843333333333 | 0.00470616666666667 |
| 7d RA | 0.130057666666667 | 0.0126965 | 0.00567716666666667 |
### Chart
| Category | H3K9me1 (8.8E-13) | H3K9me2 (5.6E-10) | H3K9me3 (5.45E-10) |
|---|---|---|---|
| ESCs | 0.232603833333333 | 0.305849 | 0.140231 |
| EBs | 0.205343833333333 | 0.396580833333333 | 0.203579833333333 |
| 1d RA | 0.188944 | 0.376187833333333 | 0.220488333333333 |
| 4d RA | 0.1787375 | 0.383358666666667 | 0.250558333333333 |
| 7d RA | 0.1758405 | 0.388025666666667 | 0.266631333333333 |
Component 2
Rel. abundance
Component 1
(C)
### Chart
| Category | ESCs | EBs | 1d RA | 4d RA | 7d RA |
|---|---|---|---|---|---|
| H3K23ac | 0.332336333333333 | 0.445563333333333 | 0.392688166666667 | 0.281313666666667 | 0.259033333333333 |
| H4K8ac | 0.131525666666667 | 0.119558666666667 | 0.1175325 | 0.0637743333333333 | 0.052848 |
| H4K12ac | 0.2086795 | 0.216775 | 0.177012666666667 | 0.0977033333333333 | 0.0784265 |
| H4K5ac | 0.0855266666666667 | 0.078839 | 0.0969016666666667 | 0.046823 | 0.0390485 |
| H3K18ac | 0.0873745 | 0.135824 | 0.118939333333333 | 0.0839721666666667 | 0.0659798333333333 |
| H3K9ac | 0.01352 | 0.0159558333333333 | 0.0162045 | 0.007693 | 0.0074795 |
| H3K14ac | 0.37446 | 0.428796666666667 | 0.45614 | 0.452924666666667 | 0.412086166666667 |
| H4K16ac | 0.304367833333333 | 0.455866333333333 | 0.536948166666667 | 0.457401833333333 | 0.448101166666667 |
### Chart
| Category | H3K27me1 (9.0E-23) | H3K27me2 (1.1E-10) | H3K27me3 (7.8E-26) |
|---|---|---|---|
| ESCs | 0.152597 | 0.522114666666667 | 0.259171666666667 |
| EBs | 0.2922685 | 0.446623666666667 | 0.0899365 |
| 1d RA | 0.2989 | 0.432383666666667 | 0.0574463333333333 |
| 4d RA | 0.308959833333333 | 0.425122166666667 | 0.055587 |
| 7d RA | 0.304044833333333 | 0.4443105 | 0.0705761666666667 |
### Chart
| Category | H3K36me1 (1.0E-04) | H3K36me2 (2.8E-10) | H3K36me3 (6.9E-11) |
|---|---|---|---|
| ESCs | 0.303771 | 0.307904833333333 | 0.0183158333333333 |
| EBs | 0.267768833333333 | 0.444017333333333 | 0.0129213333333333 |
| 1d RA | 0.284057 | 0.383830166666667 | 0.0168395 |
| 4d RA | 0.279177166666667 | 0.370694166666667 | 0.0205928333333333 |
| 7d RA | 0.272235833333333 | 0.369802 | 0.0239325 |
### Chart
| Category | |
|---|---|Rel. abundance
-Log10 ANOVA p-value
Rel. abundance
