# Supplementary material for: Histone H4 acetylation and the epigenetic reader Brd4 are critical regulators of pluripotency in embryonic stem cells
Source: BMC Genomics. 2016 Feb 4;17:95. doi: 10.1186/s12864-016-2414-y (PMC4740988; doi:10.1186/s12864-016-2414-y)
Supplement: Additional file 3: Figure S2. — Histone peptide regulation during JQ1 treatment. Heatmap of all quantified peptides using nLC-MS from histone H3 and H4 in ESCs and ESCs treated with JQ1 inhibitor. (PPTX 461 kb) [file 12864_2016_2414_MOESM3_ESM.pptx]

## Slide 1
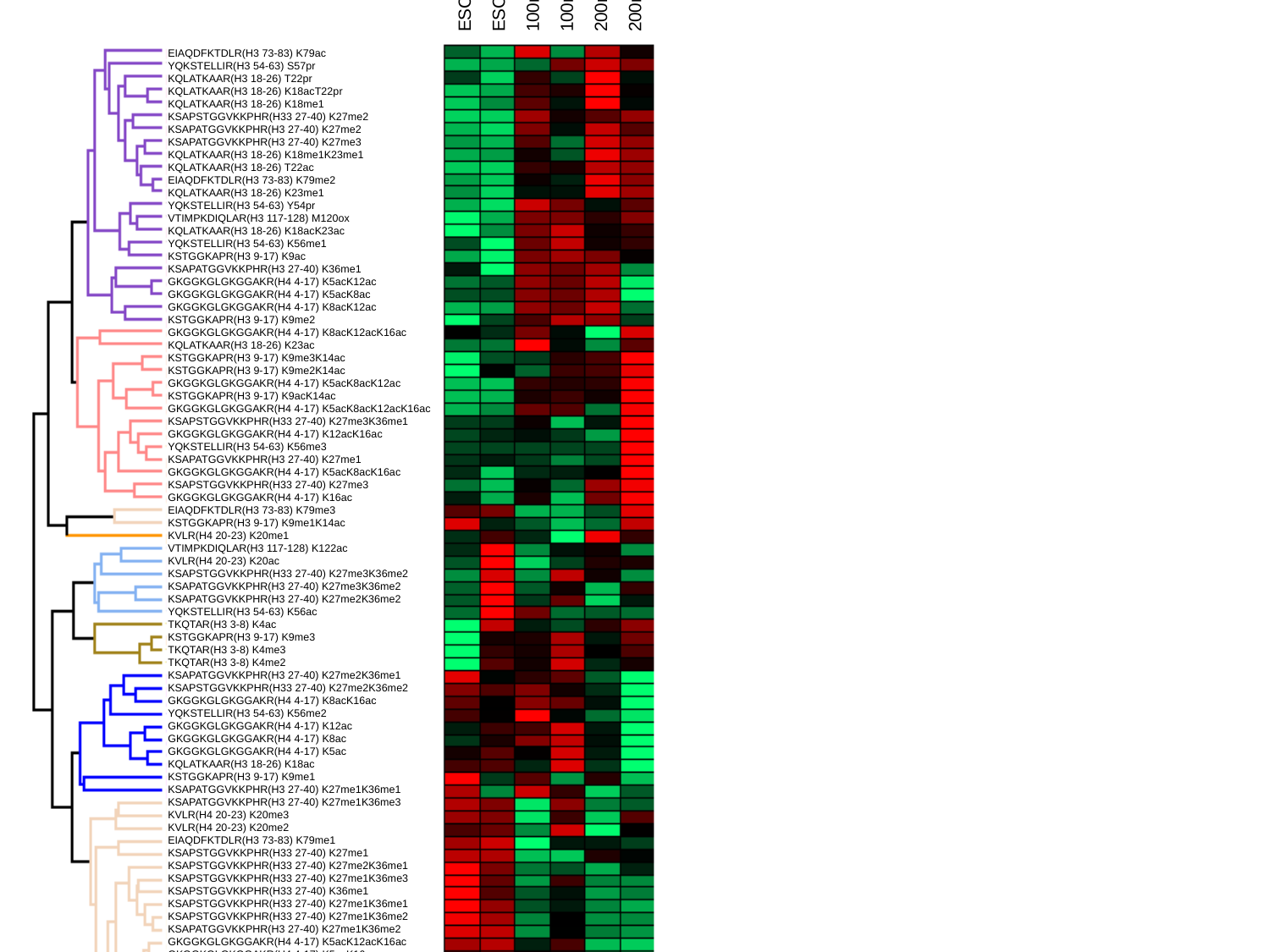

100nM JQ1 A
100nM JQ1 B
200nM JQ1 A
200nM JQ1 B
ESCs A
ESCs B
EIAQDFKTDLR(H3 73-83) K79ac
YQKSTELLIR(H3 54-63) S57pr
KQLATKAAR(H3 18-26) T22pr
KQLATKAAR(H3 18-26) K18acT22pr
KQLATKAAR(H3 18-26) K18me1
KSAPSTGGVKKPHR(H33 27-40) K27me2
KSAPATGGVKKPHR(H3 27-40) K27me2
KSAPATGGVKKPHR(H3 27-40) K27me3
KQLATKAAR(H3 18-26) K18me1K23me1
KQLATKAAR(H3 18-26) T22ac
EIAQDFKTDLR(H3 73-83) K79me2
KQLATKAAR(H3 18-26) K23me1
YQKSTELLIR(H3 54-63) Y54pr
VTIMPKDIQLAR(H3 117-128) M120ox
KQLATKAAR(H3 18-26) K18acK23ac
YQKSTELLIR(H3 54-63) K56me1
KSTGGKAPR(H3 9-17) K9ac
KSAPATGGVKKPHR(H3 27-40) K36me1
GKGGKGLGKGGAKR(H4 4-17) K5acK12ac
GKGGKGLGKGGAKR(H4 4-17) K5acK8ac
GKGGKGLGKGGAKR(H4 4-17) K8acK12ac
KSTGGKAPR(H3 9-17) K9me2
GKGGKGLGKGGAKR(H4 4-17) K8acK12acK16ac
KQLATKAAR(H3 18-26) K23ac
KSTGGKAPR(H3 9-17) K9me3K14ac
KSTGGKAPR(H3 9-17) K9me2K14ac
GKGGKGLGKGGAKR(H4 4-17) K5acK8acK12ac
KSTGGKAPR(H3 9-17) K9acK14ac
GKGGKGLGKGGAKR(H4 4-17) K5acK8acK12acK16ac
KSAPSTGGVKKPHR(H33 27-40) K27me3K36me1
GKGGKGLGKGGAKR(H4 4-17) K12acK16ac
YQKSTELLIR(H3 54-63) K56me3
KSAPATGGVKKPHR(H3 27-40) K27me1
GKGGKGLGKGGAKR(H4 4-17) K5acK8acK16ac
KSAPSTGGVKKPHR(H33 27-40) K27me3
GKGGKGLGKGGAKR(H4 4-17) K16ac
EIAQDFKTDLR(H3 73-83) K79me3
KSTGGKAPR(H3 9-17) K9me1K14ac
KVLR(H4 20-23) K20me1
VTIMPKDIQLAR(H3 117-128) K122ac
KVLR(H4 20-23) K20ac
KSAPSTGGVKKPHR(H33 27-40) K27me3K36me2
KSAPATGGVKKPHR(H3 27-40) K27me3K36me2
KSAPATGGVKKPHR(H3 27-40) K27me2K36me2
YQKSTELLIR(H3 54-63) K56ac
TKQTAR(H3 3-8) K4ac
KSTGGKAPR(H3 9-17) K9me3
TKQTAR(H3 3-8) K4me3
TKQTAR(H3 3-8) K4me2
KSAPATGGVKKPHR(H3 27-40) K27me2K36me1
KSAPSTGGVKKPHR(H33 27-40) K27me2K36me2
GKGGKGLGKGGAKR(H4 4-17) K8acK16ac
YQKSTELLIR(H3 54-63) K56me2
GKGGKGLGKGGAKR(H4 4-17) K12ac
GKGGKGLGKGGAKR(H4 4-17) K8ac
GKGGKGLGKGGAKR(H4 4-17) K5ac
KQLATKAAR(H3 18-26) K18ac
KSTGGKAPR(H3 9-17) K9me1
KSAPATGGVKKPHR(H3 27-40) K27me1K36me1
KSAPATGGVKKPHR(H3 27-40) K27me1K36me3
KVLR(H4 20-23) K20me3
KVLR(H4 20-23) K20me2
EIAQDFKTDLR(H3 73-83) K79me1
KSAPSTGGVKKPHR(H33 27-40) K27me1
KSAPSTGGVKKPHR(H33 27-40) K27me2K36me1
KSAPSTGGVKKPHR(H33 27-40) K27me1K36me3
KSAPSTGGVKKPHR(H33 27-40) K36me1
KSAPSTGGVKKPHR(H33 27-40) K27me1K36me1
KSAPSTGGVKKPHR(H33 27-40) K27me1K36me2
KSAPATGGVKKPHR(H3 27-40) K27me1K36me2
GKGGKGLGKGGAKR(H4 4-17) K5acK12acK16ac
GKGGKGLGKGGAKR(H4 4-17) K5acK16ac
KSAPATGGVKKPHR(H3 27-40) K36me2
KSAPSTGGVKKPHR(H33 27-40) K36me2
KSAPATGGVKKPHR(H3 27-40) K27me3K36me1
KSTGGKAPR(H3 9-17) K14ac
TKQTAR(H3 3-8) K4me1
Z-score
-1.5
0
1.5
