# Supplementary figures and images for: Histone H4 acetylation and the epigenetic reader Brd4 are critical regulators of pluripotency in embryonic stem cells
Source: BMC Genomics. 2016 Feb 4;17:95. doi: 10.1186/s12864-016-2414-y (PMC4740988; doi:10.1186/s12864-016-2414-y)

## Slide 1
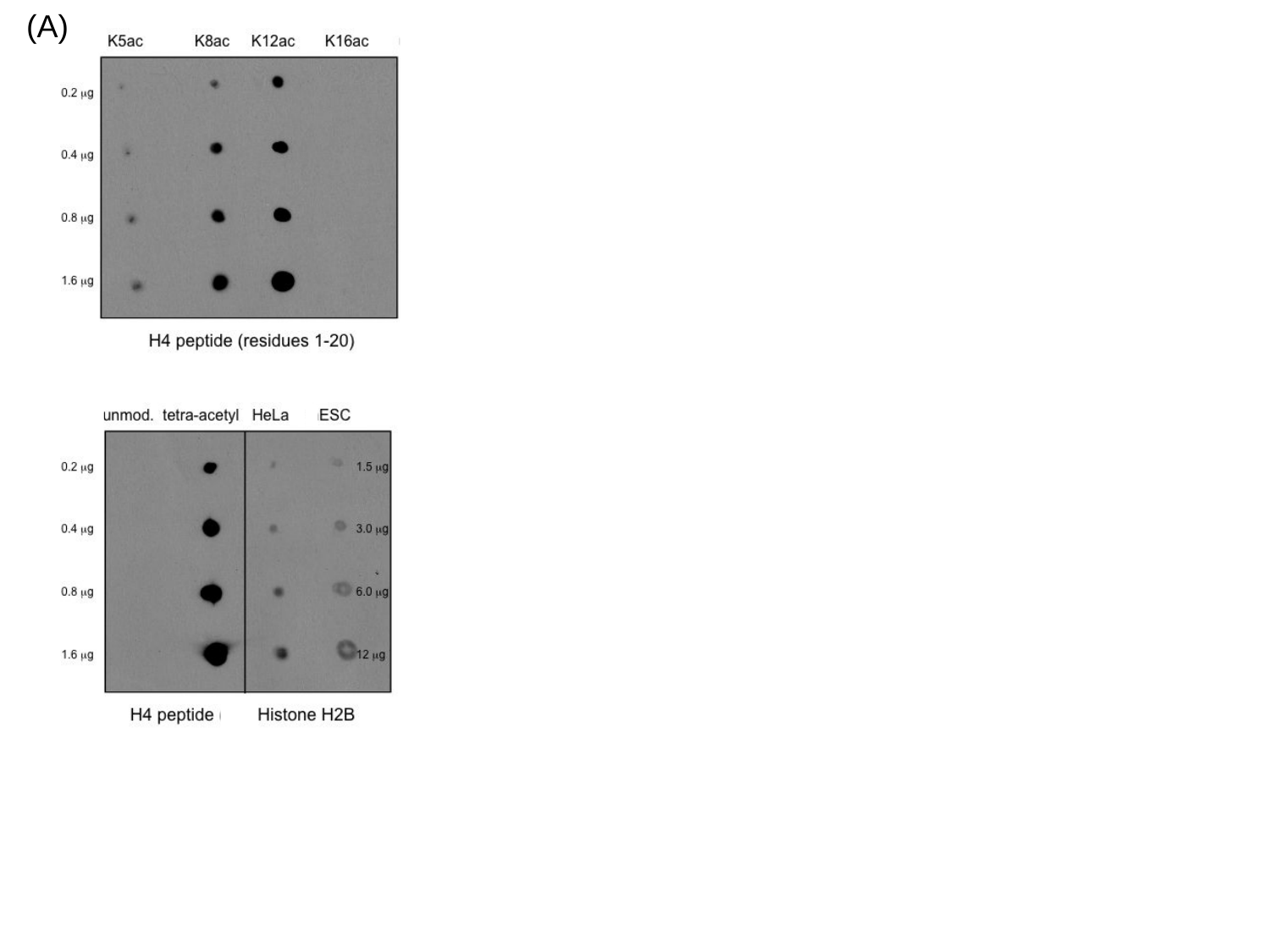

(A)

Supplement: Additional file 4: Figure S3. — Validation of antibody against histone H4 acetylation and ChIP-Seq of key chromatin binding proteins. Dot blot testing the specificity of anti-H4 acetyl antibody. Specific amounts of various H4 peptides and histone H2B were dotted onto the membrane. Antibody strongly recognizes H4 peptide singly acetylated at K8 or K12, and tetra-acetylated H4 (K5, K8, K12, K16). (PPTX 183 kb) [file 12864_2016_2414_MOESM4_ESM.pptx]

## Slide 1
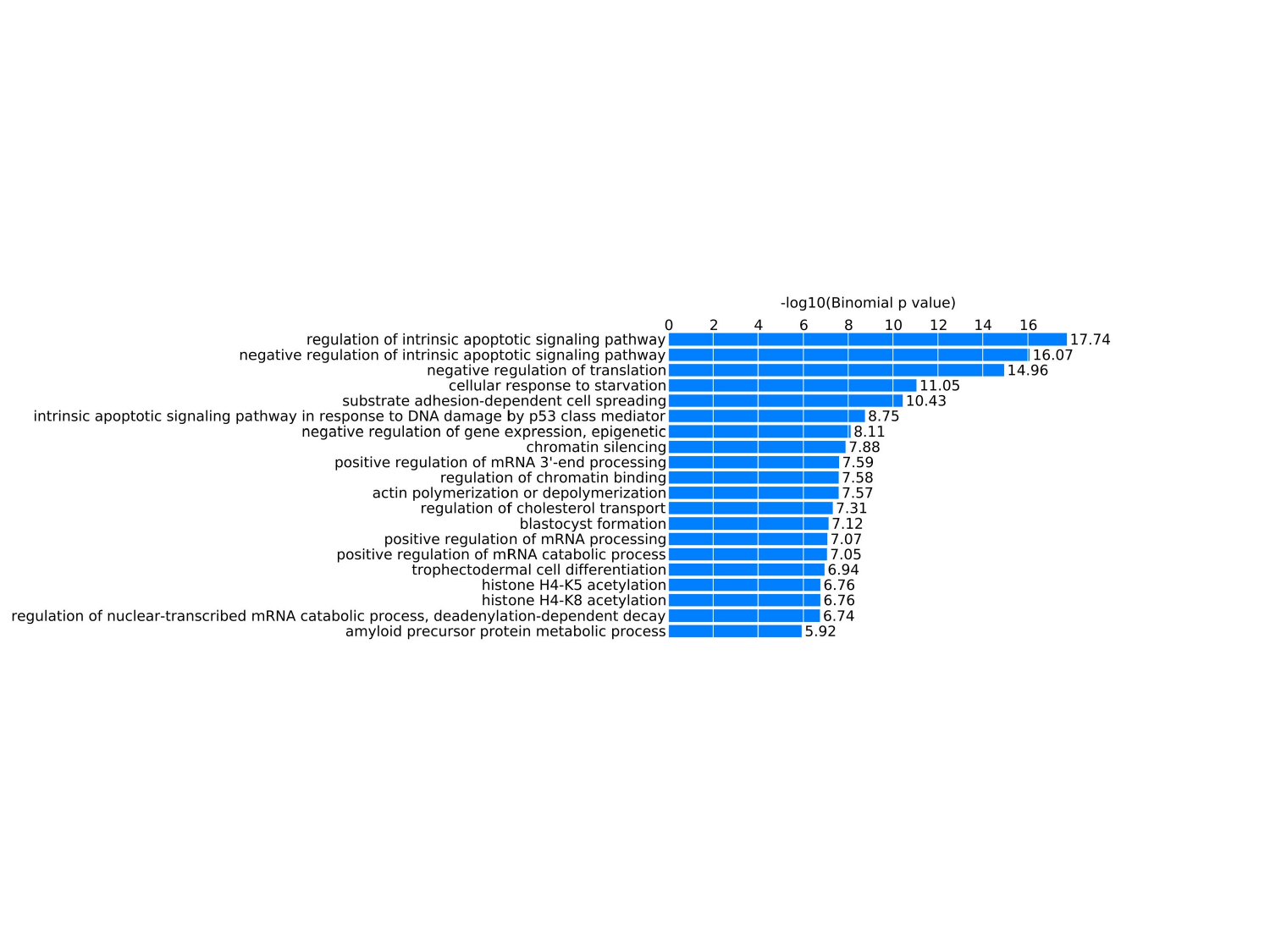

Supplement: Additional file 5: Figure S4. — GO analysis for the peak constitutive for both ESCs and EBs. (PPTX 711 kb) [file 12864_2016_2414_MOESM5_ESM.pptx]

## Slide 1
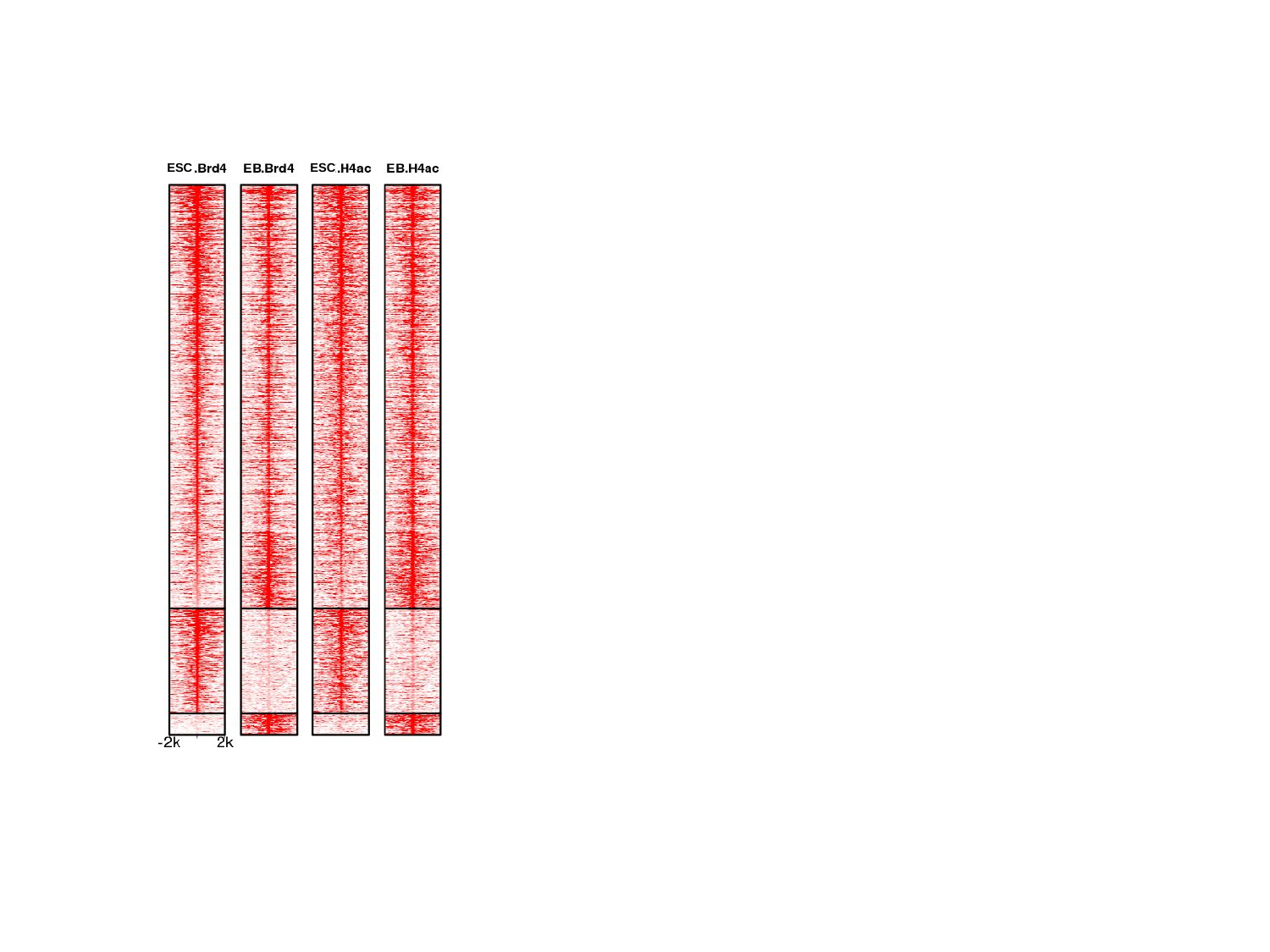

ESC
ESC

Supplement: Additional file 6: Figure S5. — Heatmap for ESC and EB specific Brd4 binding sites. Brd4 binding well matches with the enrichment for H4ac. (PPTX 526 kb) [file 12864_2016_2414_MOESM6_ESM.pptx]
